# Lomeguatrib Increases the Radiosensitivity of MGMT Unmethylated Human Glioblastoma Multiforme Cell Lines

**DOI:** 10.3390/ijms22136781

**Published:** 2021-06-24

**Authors:** Anna Kirstein, Daniela Schilling, Stephanie E. Combs, Thomas E. Schmid

**Affiliations:** 1 Institute of Radiation Medicine (IRM), Department of Radiation Sciences (DRS), Helmholtz Zentrum München, 85764 Neuherberg, Germany; anna.kirstein@helmholtz-muenchen.de (A.K.); daniela.schilling@tum.de (D.S.); stephanie.combs@tum.de (S.E.C.); 2 Department of Radiation Oncology, School of Medicine, Technical University of Munich (TUM), 81675 Munich, Germany; 3 Deutsches Konsortium für Translationale Krebsforschung (DKTK), Partner Site Munich, 81675 Munich, Germany

**Keywords:** glioblastoma, MGMT, lomeguatrib, radiosensitivity, radiotherapy

## Abstract

Background: Treatment resistance of glioblastoma multiforme to chemo- and radiotherapy remains a challenge yet to overcome. In particular, the O^6^-methylguanine-DNA-methyltransferase (MGMT) promoter unmethylated patients have only little benefit from chemotherapy treatment using temozolomide since MGMT counteracts its therapeutic efficacy. Therefore, new treatment options in radiotherapy need to be developed to inhibit MGMT and increase radiotherapy response. Methods: Lomeguatrib, a highly specific MGMT inhibitor, was used to inactivate MGMT protein in vitro. Radiosensitivity of established human glioblastoma multiforme cell lines in combination with lomeguatrib was investigated using the clonogenic survival assay. Inhibition of MGMT was analyzed using Western Blot. Cell cycle distribution and apoptosis were investigated to determine the effects of lomeguatrib alone as well as in combination with ionizing radiation. Results: Lomeguatrib significantly decreased MGMT protein and reduced radiation-induced G2/M arrest. A radiosensitizing effect of lomeguatrib was observed when administered at 1 µM and increased radioresistance at 20 µM. Conclusion: Low concentrations of lomeguatrib elicit radiosensitization, while high concentrations mediate a radioprotective effect.

## 1. Introduction

Glioblastoma multiforme (GBM) is still one of the most devastating diagnoses. Despite developments in alternative treatment options including novel chemotherapeutic agents, inhibitors, and targeted miRNA delivery, as well as extensive research in radio- and chemoresistance, improvement of patient survival is still poor. With a five-year survival of only 0.05–4.7% after diagnosis [1], mortality rates are significantly high, although the incidence rate with 0.59–3.69 cases per 100,000 persons is relatively low [1]. GBM is a grade IV diffuse astrocytic tumor and characterized by diffuse infiltration and uncontrolled cellular proliferation [2]. According to the World Health Organization (WHO) classification of tumors of the central nervous system, GBM is subdivided into isocitrate dehydrogenase (IDH)-wildtype or primary GBM, IDH-mutant, or secondary GBM and not otherwise specified (NOS) GBM [3]. A total of 90% of all glioblastoma are primary GBM, with a median overall survival of 15 months and a median age at diagnosis of 62 years [3]. It develops de novo within 3–6 months from glial progenitor cells [4]. Secondary GBM, in contrast, is less common with a median overall survival of 31 months and a median age at diagnosis of 44 years [3]. Originating from low-grade astrocytomas (WHO grade II) and anaplastic astrocytomas (WHO grade III), secondary GBM develops over several years [4]. Primary and secondary GBM are histologically very similar and can only be distinguished by their unique mutation patterns [5]. In case of an inconclusive IDH status analysis, GBM is classified as NOS glioblastoma.

Magnetic resonance imaging (MRI) serves as the first tool of diagnosis, however, a biopsy and pathological examination are required to confirm GBM and determine the subtype. First in line for the treatment is the maximal safe surgical resection of the tumor to relieve symptoms caused by the increased intracranial pressure such as headaches, nausea, vomiting, somnolence, and visual impairments [5]. Due to the high invasive potential as well as extensive vascularization into the surrounding brain tissue complete resection is almost impossible and is often the cause of tumor recurrence [2,5]. Therefore, the extent of resection (EOR) is a crucial predictor for treatment outcome, as it has been postulated an EOR of about 98% is required to attain prolonged survival without increasing postoperative neurological morbidities [6]. Longest life expectancies are achieved when surgical resection is followed by radiation therapy and chemotherapy.

Since 2005, the chemotherapeutic agent temozolomide (TMZ) is orally administered with a daily dose of 75 mg per m^2^ for five consecutive days for six weeks [7]. Conventional radiotherapy is given in 30 fractions at 2 Gy over six weeks to a total dose of 60 Gy [7] precisely to the tumor resection cavity. TMZ is administered for six more cycles at 150–200 mg per m^2^ for maintenance [8]. After a median time of 32–36 weeks, recurrence or progression is expected with a mortality rate of about 100% [9].

TMZ is an alkylating agent methylating several sites within the DNA: one site in the base adenine is the N^3^ position and two positions of the base guanine are N^7^ (70%) and O^6^ (5%) [10,11]. Only the latter mentioned site, creating the base O^6^-methylguanine (O^6^-MG), is assumed to have cytotoxic and mutagenic potential [12,13]. During DNA replication O^6^-MG creates a wobble base pair with thymine, which is recognized by the mismatch repair (MMR) pathway. MMR excises the mismatched thymine but will subsequently replace it with another thymine. These futile circles of thymine deletion and insertion will eventually lead to a depletion of deoxythymidine triphosphates (dTTP) resulting in lack of DNA synthesis and ultimately cell death by apoptosis [14]. The O^6^-methylguanine-DNA-methyltransferase (MGMT) specifically removes these methyl adducts from the O^6^-MG preventing futile thymine deletion and insertion circles [15]. The methyl group is transferred to the cysteine residue Cy 145 in the active site of MGMT [16]. The resulting alkylthioether cannot be regenerated and MGMT gets ubiquitinated and subsequently degraded [15]. MGMT is thus called a suicide enzyme. Patients receiving TMZ and showing an unmethylated MGMT promoter region, hence, have only little benefit from TMZ treatment, as MGMT counteracts the therapeutic efficacy of TMZ [17]. The promoter methylation status of the MGMT gene is nowadays evaluated in every GBM patient to predict chemotherapy outcomes [17]. However, the role of MGMT during radiotherapy is not fully understood. Therefore, it is of great importance to find new and personalized treatment options for MGMT unmethylated patients during radiotherapy to omit or overcome TMZ resistance and improve overall survival.

O(6)-(4-bromothenyl)guanine also known as PaTrin-2, lomeguatrib, O6BTG, or 4BTG is a potent MGMT inhibitor first synthesized by McElhinney et al. [18]. By modifying the O^6^ position with heterocyclic moieties they synthesized guanine derivatives compatible with the stereochemical requirements at the MGMT’s active site. First characterization experiments in vivo were performed by Middleton et al. [19]. They observed MGMT depletion in various normal tissue organs as well as in subcutaneous melanoma tumor xenografts for up to 24 h after a single dose of 20 mg per kg^−1^ lomeguatrib [19].

Therefore, this work aimed to investigate the effects of lomeguatrib on radiosensitivity of GBM cell lines with an unmethylated MGMT promoter region. Other cellular processes such as apoptosis, cell cycle distribution, and DNA repair were investigated as well.

## 2. Results

### 2.1. T98G Is more Radioresistant Than LN18 and U118

Colony-forming assay (CFA) was performed in order to determine the radiosensitivity of established human glioblastoma multiforme cell lines. Cells were pre-plated in 12-well plates 24 h prior to 0 Gy, 1 Gy, 2 Gy, 4 Gy, 6 Gy, and 8 Gy ionizing radiation.

**Figure 1:**
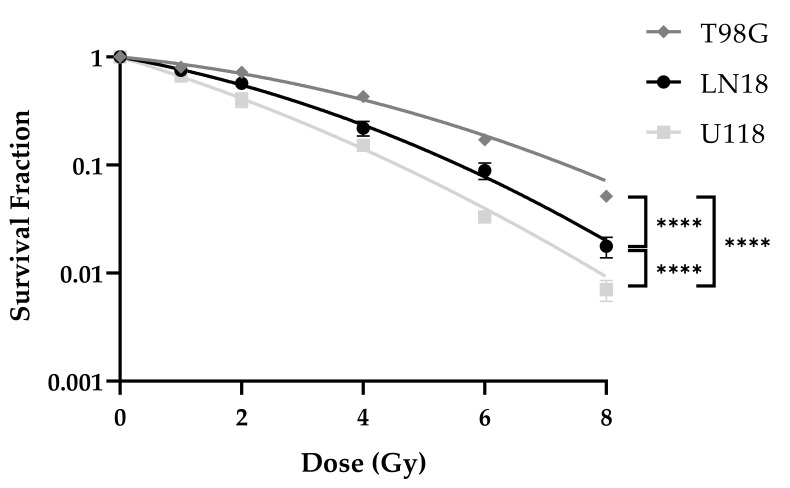
Colony-forming assay (CFA) of LN18, T98G, and U118 cell lines. Survival curves were fitted to the linear-quadratic model. Curves present the mean values of at least three replicates and error bars show the standard error of the mean. (Two-Way ANOVA; **** *p* ≤ 0.0001).

T98G was the most radioresistant cell line with a D_50_ of 3.30 Gy, while LN18 was intermediate radiosensitive with a D_50_ value of 2.26 Gy, while U118 was the most radiosensitive amongst the three tested cell lines with a D_50_ of 1.66 Gy. Significant differences in the survival curves were detected between LN18 and T98G (*p* < 0.0001), between LN18 and U118 (*p* < 0.0001), and between T98G and U118 (*p* < 0.0001) cell lines (Figure 1).

### 2.2. Lomeguatrib Decreases MGMT Protein Levels

**Figure 2:**
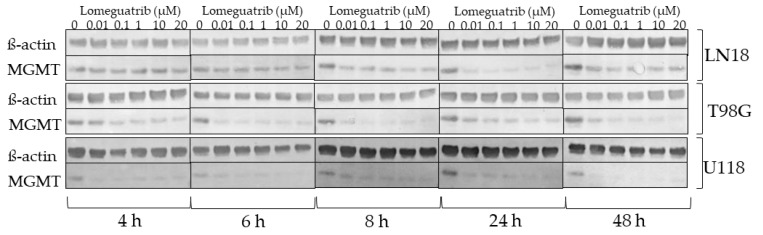
Western Blot analysis of LN18, T98G, and U118 cell lines. Increasing concentrations of lomeguatrib were added for 4 h, 6 h, 8 h, 24 h, and 48 h. Shown are the representative blots for the protein levels of MGMT and ß-actin.

In order to determine optimal conditions for MGMT inhibition, the effect of different lomeguatrib concentrations and time points on MGMT protein levels were investigated. The three established human glioblastoma, MGMT promoter unmethylated cell lines (LN18, T98G, and U118) were exposed to different lomeguatrib concentrations for 4, 6, 8, 24, or 48 h and lysates were subjected to Western Blot analysis (Figure 2).

Already 4 h after lomeguatrib treatment a decrease in MGMT protein was observed in T98G and U118 cell lines at all tested concentrations. In LN18 cells MGMT inhibition became visible after 24 h of lomeguatrib treatment at all concentrations. Inhibition of MGMT was still detectable after 48 h. Since MGMT inhibition was visible after 24 h in all cell lines, we decided to use this treatment duration (24 h) for further experiments.

### 2.3. High Dose Lomeguatrib Changes Cell Cycle Distribution

**Figure 3:**
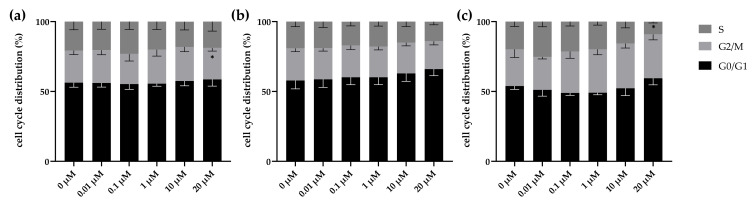
Cell cycle distribution was altered by lomeguatrib in all cell lines. (**a**) shows LN18, (**b**) shows T98G, and (**c**) shows U118 cell cycle distribution 24 h after lomeguatrib addition. Bars present the mean values and error bars the standard error of the mean of at least three replicates. (Student’s *t*-test; * *p* ≤ 0.05).

With the aim to determine whether lomeguatrib affects cell cycle distribution, cell cycle analysis was performed 24 h after lomeguatrib treatment. In LN18 cells a significantly decreased G2/M fraction (*p* = 0.0197) was detected at the highest concentration of 20 µM as well as a trend towards an increased G1 fraction (*p* = 0.0562) at 20 µM lomeguatrib, compared to the untreated sample (Figure 3a). No effect was detected upon lomeguatrib treatment in the T98G cell line (Figure 3b). A significantly decreased S phase (*p* = 0.0411) was detected in the U118 cell line at 20 µM lomeguatrib, compared to the untreated sample (Figure 3c).

### 2.4. Lomeguatrib Does Not Affect Cell Proliferation

To determine the effects of lomeguatrib on cell proliferation the alamarBlue proliferation assay upon 1 µM and 20 µM lomeguatrib treatment for 24 h was performed. LN18 and T98G were comparable fast proliferating cell lines with doubling times of 16.4 h ± 5.4 h, and 16.1 h ± 1.8 h respectively, while U118 was slower proliferating with a doubling time of 20.1 ± 4.8 h (Table 1). Neither 1 µM lomeguatrib nor 20 µM lomeguatrib significantly changed the doubling times of any tested cell line.

### 2.5. Irradiation Does Not Change MGMT Protein Levels

In order to investigate if irradiation in combination with lomeguatrib affects protein levels of MGMT, Western Blot analysis was performed. Cells were treated with different lomeguatrib concentrations for 24 h, then irradiated with 0 Gy or 8 Gy and 24 h later lysates were prepared.

**Figure 4:**
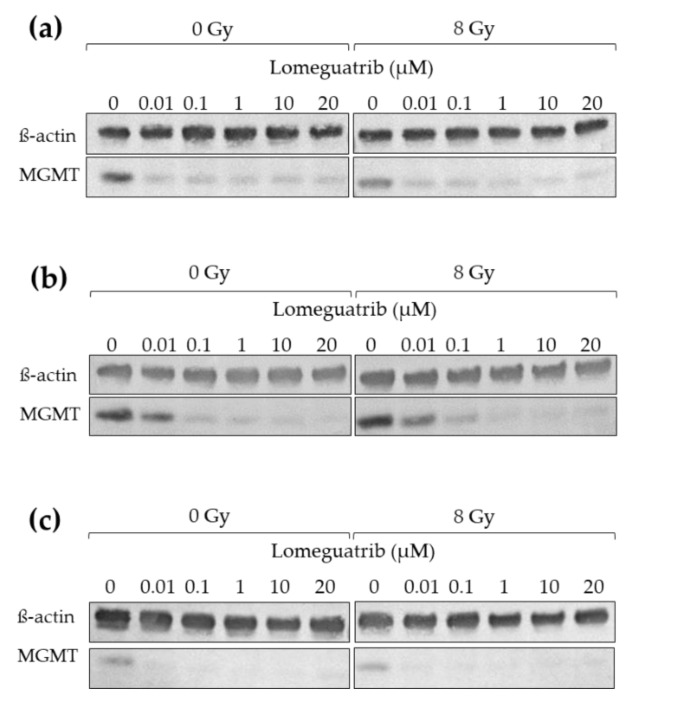
Western Blot analysis of (**a**) LN18, (**b**) T98G, and (**c**) U118 cell lines. Increasing concentrations of lomeguatrib were added for 24 h before 0 Gy or 8 Gy irradiation. Lysates were prepared 24 h after irradiation. Shown are the representative blots for MGMT and ß-actin.

Neither 8 Gy ionizing radiation alone nor combined with increasing concentrations of lomeguatrib does change MGMT protein levels compared to the unirradiated controls in any of the tested cell lines (Figure 4).

### 2.6. Lomeguatrib Exhibits a Radiosensitizing Effect Only at Low Doses

**Figure 5:**
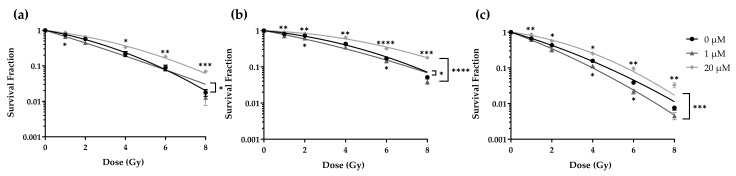
Colony-forming assay (CFA) of (**a**) LN18, (**b**) T98G, and (**c**) U118 cell lines. Survival curves are fitted to the linear-quadratic model. Curves present the mean values of at least three replicates and error bars show the standard error of the mean. Stars below the curve indicate significances between 0 µM and 1 µM, stars above the curve indicate significances between 0 µM and 20 µM calculated using Student’s *t*-test. Stars behind the curves indicate differences between 0 µM and 1 µM or 0 µM and 20 µM survival curves determined using Two-Way ANOVA. (* *p* ≤ 0.05, ** *p* ≤ 0.01, *** *p* ≤ 0.001, **** *p* ≤ 0.0001).

To determine whether lomeguatrib affects the radiosensitivity of GBM cell lines cells were treated with lomeguatrib for 24 h before irradiation and clonogenic survival was assessed. Treatment with 1 µM or 20 µM lomeguatrib changed the cell survival fraction in a dose-dependent manner. Treatment with 1 µM decreased the radioresistance of LN18 (Figure 5a), T98G (Figure 5b), and U118 (Figure 5c) cells. In contrast, treatment with 20 µM lomeguatrib increased the radioresistance in comparison to the untreated controls.

Two-Way ANOVA was used to calculate differences between the treatment groups. Increased radiosensitivity was observed in LN18 (*p* = 0.0126) and T98G (*p* = 0.0150) upon treatment with 1 µM lomeguatrib, while in U118 cells only a non-significant increased radiosensitivity was observed (*p* = 0.1468). Radioresistance was increased in T98G (*p* < 0.0001) and U118 (*p* = 0.0008) upon 20 µM lomeguatrib treatment, and a trend towards an increased radioresistance was observed in LN18 cells (*p* = 0.0954). Differences between 0 µM and 1 µM as well as 0 µM and 20 µM for each radiation dose were calculated using Student’s *t*-test and are indicated in Figure 5 below (1 µM) and above (20 µM) the survival curves.

D_50_ values of the LN18 cell line increased from 2.27 ± 0.24 Gy (untreated) to 3.05 ± 0.04 Gy upon 20 µM lomeguatrib (*p* = 0.045) but did not change upon 1 µM lomeguatrib (Table 2). For the T98G cell line, D_50_ values increased from 3.29 ± 0.12 Gy of the untreated cells to 4.72 ± 0.08 Gy upon 20 µM lomeguatrib (*p* = 0.001) and decreased to 2.54 ± 0.18 Gy (*p* = 0.030) in the presence of 1 µM lomeguatrib (Table 2). D_50_ values increased from 1.67 ± 0.11 Gy to 2.51 ± 0.20 Gy (*p* = 0.008) upon 20 µM lomeguatrib in U118 cells and remained unchanged upon 1 µM lomeguatrib treatment (Table 2). The Sensitization Enhancement Ratio 50% (SER) indicates the extent of radiosensitization. Values greater than 1 indicate a radiosensitizing effect, while values lower than 1 indicate greater radioresistance. As seen in Table 2, treatment with 1 µM lomeguatrib resulted in a significantly increased SER (1.36 in LN18, *p* = 0.012; 1.30 in T98G, *p* = 0.005; and 1.35 in U118, *p* = 0.026), while 20 µM lomeguatrib treatment resulted in a significant decrease in SER in all cell lines (0.76 in LN18, *p* = 0.017; 0.70 in T98G, *p* < 0.0001; and 0.66 in U118, *p* = 0.007).

### 2.7. High Dose Lomeguatrib Decreases Radiation-Induced G2/M Arrest

Next, we investigated the combined effect of lomeguatrib and radiation on cell cycle distribution. Cells were treated with lomeguatrib for 24 h before irradiation and were fixed 24 h later.

**Figure 6:**
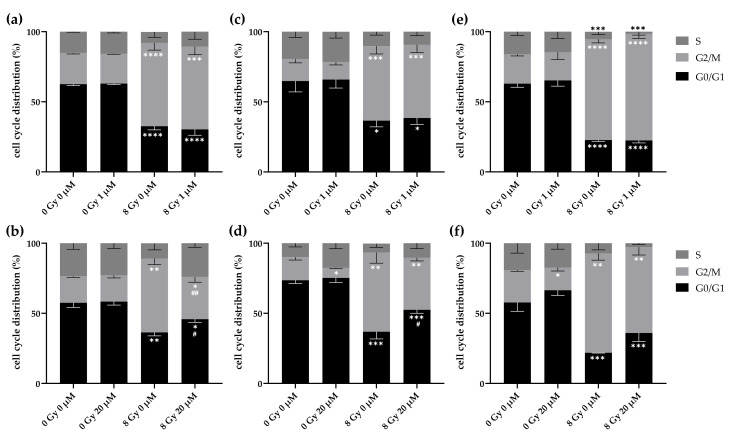
G2/M cell cycle phase was significantly decreased by 8 Gy ionizing radiation combined with 20 µM lomeguatrib. (**a**) and (**b**) show LN18, (**c**) and (**d**) show T98G, and (**e**,**f**) show U118 cell cycle distribution 24 h after irradiation. Bars present the mean values, and error bars the standard deviation of at least three replicates. Asterisks indicate significances of the different treatments versus the 0 Gy 0 µM lomeguatrib sample of the respective cell cycle phase (Student’s *t*-test; * *p* ≤ 0.05, ** *p* ≤ 0.01, *** *p* ≤ 0.001, **** *p* ≤ 0.0001), while hash symbols represent significances between 0 Gy 0 µM to 0 Gy 1 µM or 20 µM and 8 Gy 0 µM to 8 Gy 1 µM or 20 µM.(Student’s *t*-test; # *p* ≤ 0.05, ## *p* ≤ 0.01).

8 Gy ionizing radiation alone significantly enhanced G2/M cell cycle fraction (LN18: *p* < 0.0001; T98G: *p* = 0.0003; U118: *p* < 0.0001). Treatment with 1 µM without irradiation did not change cell cycle distribution in any of the tested cell lines (Figure 6a,c,e and Table 3). 1 µM lomeguatrib in combination with 8 Gy irradiation did not show any effect on cell cycle distribution as well, compared to the untreated sample or the 8 Gy irradiated sample without lomeguatrib. In contrast, 20 µM lomeguatrib alone decreased G2/M phase in T98G cells (Figure 6d, *p* = 0.0140), while in the other cell lines no difference was detected. 8 Gy irradiation combined with 20 µM lomeguatrib decreased G2/M phase in LN18 cells (*p* = 0.0085) and increases G1 phase accordingly (*p* = 0.0332) with a trend towards a decreased S phase (*p* = 0.0687) compared to the 8 Gy irradiated sample without lomeguatrib. An increased G1 phase (*p* = 0.0342) and a trend towards a decreased G2/M phase (*p* = 0.0511) in the 8 Gy irradiated and 20 µM lomeguatrib treated sample was observed in T98G as well, compared to the 8 Gy irradiated sample. In U118 cells, no significant difference was detected comparing the 8 Gy irradiated sample to the 8 Gy and 20 µM lomeguatrib treated sample, with only a trend towards an increased G1 phase (*p* = 0.0809).

In summary, these data indicate that lomeguatrib counteracts the radiation-induced G2/M arrest.

All *p*-values comparing each dose in each cell cycle phase for all three cell lines were calculated using Student’s *t*-test and are presented in Table 3. Significant differences are highlighted in bold.

### 2.8. Lomeguatrib Does Not Affect Radiation-Induced Apoptosis

**Figure 7:**
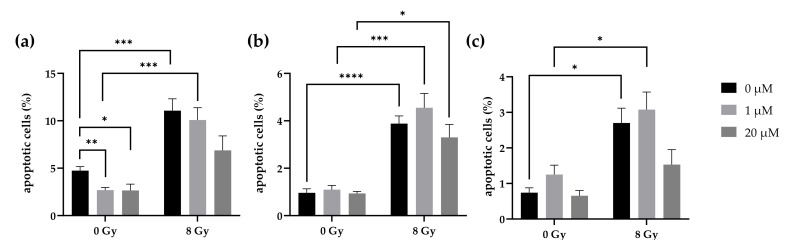
Apoptosis in (**a**) LN18, (**b**) T98G cells, and (**c**) U118 cells upon lomeguatrib and radiation treatment. Bars present the mean values and error bars the standard error of the mean of at least three replicates. (Student’s *t*-test; * *p* ≤ 0.05, ** *p* ≤ 0.01, *** *p* ≤ 0.001, **** *p* ≤ 0.0001).

The effects of lomeguatrib in combination with radiation on apoptosis were measured 48 h after irradiation via caspase-3/7 FACS analysis. 1 µM, as well as 20 µM alone, decreased apoptotic cells in the LN18 cell line (*p* = 0.0025 and *p* = 0.0296, Figure 7a). However, no change was observed in T98G (Figure 7b) and U118 (Figure 7c) cells. 8 Gy ionizing radiation alone (LN18: *p* = 0.0007; T98G: *p* < 0.0001; U118: *p* = 0.0135), as well as in combination with 1 µM lomeguatrib (LN18: *p* = 0.0002; T98G: *p* = 0.0003; U118: *p* = 0.0104) increased apoptotic cell fraction in all cell lines. 20 µM lomeguatrib combined with 8 Gy radiation did not change apoptotic cell fraction in LN18 and U118 cells, however, an increase was observed in T98G cells (*p* = 0.0125).

## 3. Discussion

Up to date, the diagnosis of glioblastoma multiforme results in most cases in death during the first 15 months. Despite advances in finding predictive biomarkers, such as MGMT promoter methylation status, the five-year survival is still less than 3%, making GBM the deadliest of all cancers [1]. Although MGMT promoter methylation is favorable in the course of temozolomide therapy [17], advances for MGMT unmethylated patients have not yet been applied to the daily routine treatment. In recent years, several approaches were tested in clinical trials to circumvent MGMT expression and MGMT protein levels on post-translational levels, such as O^6^-benzylguanine [20,21,22], PARP inhibitors [23,24,25], as well as miRNAs [26,27,28]. However, none of these approaches have proven beneficial for routine GBM treatment, yet.

Lomeguatrib is a highly specific and highly potent MGMT inhibitor that was specifically designed to inactivate MGMT protein and to prevent severe side effects, such as myelosuppression as observed during the administration of O^6^-benzylguanine. Here, we investigated the effects of lomeguatrib treatment in combination with ionizing radiation on MGMT unmethylated human glioblastoma multiforme cell lines.

The choice of lomeguatrib concentration in vitro was based on Western Blot analysis, where different concentrations of lomeguatrib were tested for different time points (Figure 2). We could clearly demonstrate that already concentrations as low as 0.01 µM lomeguatrib could reduce MGMT protein levels by 60–80% after 6 h and 8 h (Figure 2). These findings are in accordance with Reinhard et al. [29] who determined an IC_50_ of 0.004 µM in HeLa S3 cervix adenocarcinoma cells, as well as Clemons et al. [30] who calculated an IC_50_ of 0.006 µM after 2 h of lomeguatrib treatment in MCF-7 breast adenocarcinoma cells. As other papers showed a significant decrease in MGMT protein after higher concentrations of 20 µM [31] or 50 µM [32,33], we decided to use 1 µM as well as 20 µM for 24 h before irradiation treatment in all further experiments.

Since all of the published works characterized the effect of lomeguatrib in combination with TMZ, our interest to combine lomeguatrib with ionizing radiation presents a completely new approach.

First, we could show, that lomeguatrib alone neither affected cell cycle distribution (Figure 3), nor cell proliferation (Table 1). These findings are well in line with previous findings from Taspinar et al. [32] and Ugur et al. [33], who could not detect a difference in cell cycle distribution 72 h after 50 µM lomeguatrib treatment in human glioblastoma multiforme and human anaplastic astrocytoma cell lines. Further, Clemons et al. [30] showed that upon 0.006 µM lomeguatrib treatment no growth inhibitory effect was observable. Signorell et al. [34] tested various concentrations of lomeguatrib and were able to find reduced cell viability in higher lomeguatrib concentrations of 20 µM and 40 µM, but not in lower concentrations ranging from 1.25 µM to 10 µM.

Interestingly, the combination of 1 µM or 20 µM with 8 Gy ionizing radiation did not change the effect of lomeguatrib on MGMT protein inhibition (Figure 4).

One important determinant for sensitivity to radiation is cell cycle regulation, with the G2/M phase being the most radiosensitive, and the G1 and S phase the less radiosensitive phases [35]. Here, we could show that 8 Gy ionizing radiation increased the cell fraction in the G2/M phase. It is long known that cells are arrested at the G2 checkpoint upon DNA damage caused by ionizing radiation to enable DNA repair and to prevent entering mitosis [36]. While lomeguatrib at a lower concentration of 1 µM did not affect cell cycle distribution (Figure 6), 20 µM lomeguatrib alone decreased G2/M phase fraction in two cell lines (T98G and U118). The radiation-induced G2/M arrest is decreased by lomeguatrib in all cell lines (Figure 6) with a subsequent increase in G1 and S cell cycle fraction. One possible explanation is a radioprotective property of lomeguatrib at higher concentrations, preventing DNA damage seen in a reduced G2/M fraction.

A different possibility is the interaction of lomeguatrib with key regulators of the G2/M cell cycle checkpoint. The G2/M checkpoint, which is the DNA damage checkpoint, is mainly regulated by CyclinB-Cdc2 activity. The CyclinB-Cdc2 complex in its phosphorylated and therefore inactive form prevents G2/M cell cycle progression [37]. The initiation of a positive feedback loop activating the phosphatase Cdc25 dephosphorylates Wee1 and Myt1, which inhibit the Cdc2-Cyclin B complex [37]. This activation and accumulation of the CyclinB-Cdc2 complex follow the all-or-none response eventually promoting entry into mitosis [38]. It could be hypothesized, that lomeguatrib not only inhibits MGMT protein but also cyclin B or Cdc2 directly, preventing the accumulation of the complex necessary to initiate mitosis, arresting the cells in the G2/M phase. Since Wee1 and Myt1 are CyclinB-Cdc2 inhibitors an upstream overexpression might be possible as well.

Another important checkpoint during cell cycle progression is the G1/S checkpoint. Natural withdrawal from cell cycle progression only happens upon growth-factor deprivation or from growth-inhibitory signals in early-to-mid G1 phase [39]. The responsible checkpoint is controlled by pRb (retinoblastoma protein)/E2F (transcription factor) and admits the cells into DNA replication and cell division. In its active form, pRb binds to the transcription factor E2F thereby inhibiting E2F from binding to promoter regions coding for necessary proteins required for S phase transition [40]. Therefore, pRb prevents cell cycle progression, and only its inactivation via phosphorylation leads to cell cycle progression beyond G1 phase [40]. This phosphorylation of pRb is mediated by the cyclin E:CDK2 and cyclin D:CDK4/6 complexes [40]. Accordingly, pRb functions as a tumor suppressor gene but is dysfunctional in many cancer types [39]. In most glioblastoma multiforme a dysregulation of the pRb signaling pathway is observed, as well as CDK4/6 amplification [41] leading to a dysfunctional cell cycle transition from G1 to S phase. Since a growth inhibitory effect was observed upon a high concentration of lomeguatrib, as well as a G1 cell cycle arrest, it can be hypothesized that lomeguatrib also inhibits CDK4/6 leading to a lack of cyclin D:CDK4/6 complexes unable to phosphorylate pRb that eventually leads to a G1 cell cycle arrest.

Due to a decrease of cells in the radiosensitive G2/M phase and the subsequent accumulation in the radioresistant G1 and S cell cycle phases upon combined irradiation and 20 µM lomeguatrib treatment, enhanced radioresistance in the clonogenic survival assay was assumed. Clonogenic survival means a cell has survived a given dose of radiation or inhibitor treatment and has retained its reproductive integrity to divide indefinitely to form colonies [42]. As seen in Figure 5 our assumption proved correct, and cells treated with 20 µM lomeguatrib before ionizing radiation treatment became more radioresistant compared to the untreated cells. This could be due to the accumulation of cells in the radioresistant G1 and S cell cycle phase caused by the higher lomeguatrib concentration, which here acts as a radioprotector. However, a radiosensitizing effect was observed after 1 µM lomeguatrib treatment, which cannot be explained by cell cycle data.

Upon the loss of a cells’ reproductive integrity, cells will ultimately die. The dominant mechanism of cell death following ionizing radiation besides necrosis and apoptosis is mitotic catastrophe during cell division [42]. However, radiation-induced apoptosis can be important as mitotic catastrophe, in order to improve radiotherapy [42]. In contrast to the findings from Taspinar et al. [32] and Shi et al. [43], who detected an increase in apoptotic cells following lomeguatrib treatment in glioblastoma, respectively in pancreatic cancer cell lines, we observed a decrease in apoptotic cells after 1 µM and 20 µM lomeguatrib treatment in the LN18 cell line. Further, we were able to detect induction of apoptosis after combined treatment of 1 µM lomeguatrib with 8 Gy ionizing radiation, while the combination of 8 Gy and 20 µM lomeguatrib did not change the rate of apoptotic cells. Since Shi et al. neither specified lomeguatrib concentration nor treatment duration, it is possible that even higher concentrations of lomeguatrib might be necessary to induce apoptosis, as Taspinar et al. have demonstrated. In their work, they analyzed apoptosis upon 50 µM lomeguatrib in the G1 sub-population and reported a significant increase in apoptotic cell death. Combination with ionizing radiation might also increase radiation-induced apoptosis but has yet to be tested. As we were not able to detect induction of apoptosis upon lomeguatrib treatment alone, it might be possible that lomeguatrib exerts its radiosensitizing effect in the lower concentrations via a different cell death pathway, such as mitotic catastrophe or necrosis.

In summary, we could clearly demonstrate that lomeguatrib significantly inactivated MGMT protein in the three tested MGMT promoter unmethylated human glioblastoma cell lines already at low concentrations and short treatment times (>6 h). Lomeguatrib showed a radiosensitizing effect at lower concentrations and an increased radioresistant effect at higher concentrations. Further, higher concentrations of 20 µM reduced the G2/M cell population in combination with 8 Gy ionizing radiation and increased the G1 and S phase cell fraction accordingly. The underlying mechanism has yet to be investigated and we propose an interaction between lomeguatrib and the key regulators of the G1-to-S or G2-to-M transition point. As DNA damage plays a crucial role in the efficacy of radiotherapy the effect of lomeguatrib on DNA double-strand breaks, as well as subsequent cell death needs further investigation; here we propose a strong dose-dependent effect of lomeguatrib.

This study presents new insights on the strong dose-dependent effects of lomeguatrib in vitro, which could help to minimize myelosuppression and hematologic side effects observed with other MGMT inhibitors, such as O6-benzylguanine [21,22]. We are the first to show a beneficial combination of lomeguatrib with ionizing radiation treatment. However, further in vivo investigations and validations are necessary to confirm these findings and to improve and establish new treatment options for MGMT unmethylated glioblastoma multiforme with the combination of lomeguatrib and ionizing radiation.

## 4. Materials and Methods

### 4.1. Cell Lines and Culture Conditions

Established human glioblastoma multiforme cell lines were obtained from the University Hospital of Heidelberg (Heidelberg, Germany; LN18) or purchased from the American Type Culture Collection (Manassas, VA, USA; T98G and U118). Cells were regularly checked for the absence of mycoplasma and cell line authentication was performed by Eurofins Genomics. LN18 and U118 were cultured in high glucose DMEM (Sigma-Aldrich, St. Louis, MO, USA) and T98G in low glucose DMEM (Sigma-Aldrich, St. Louis, MO, USA) supplemented with 10% FCS, 100 U mL^−1^ penicillin, and 100 U mL^−1^ streptomycin (Sigma-Aldrich, St. Louis, MO, USA). All cell lines were maintained in a humidified atmosphere of 5% CO_2_ at 37 °C. Cells were seeded 24 h before lomeguatrib treatment i.e., 48 h before irradiation treatment.

### 4.2. Lomeguatrib and Radiation Treatment

Lomeguatrib was purchased from MedChemExpress LLC (Princeton, NJ, USA) and dissolved in DMSO (Sigma-Aldrich, St. Louis, MO, USA). The stock solution of 6.13 mM was stored at −80 °C and diluted at 1:10 or 1:100 in medium immediately before use. Final DMSO concentrations did not exceed 0.3%. Cells were seeded 24 h before lomeguatrib treatment for 24 h before irradiation.

X-ray irradiation was performed at an RS225A irradiation device (Gulmay, XStrahl, Camberley, UK) at a dose rate of 0.9 Gy min^−1^ at 15 mA and 200 kV with a 0.5 mm copper filter and a distance to the x-ray tube of 15 cm.

### 4.3. Colony-Forming Assay (CFA)

Radiosensitivity of all cell lines was determined using the clonogenic survival assay. Cells were pre-plated at appropriate cell numbers per dose per 12-well plate and treated with 0 µM, 1 µM, or 20 µM lomeguatrib 24 h prior to 0 Gy, 1 Gy, 2 Gy, 4 Gy, 6 Gy, and 8 Gy irradiation. Followed by an 8-day (T98G and U118) respectively 12-day (LN18) incubation period, colonies were fixed with 100% −20 °C cold methanol and stained using 0.1% crystal violet. Colonies consisting of at least 50 cells were manually counted as one colony. Plating efficiencies, as well as survival fractions, were calculated in order to plot survival curves fitted to the linear-quadratic model:

(1) $$ln\;SF=-\alpha \times D-ß\times D^{2}$$

α and ß values were derived from the linear-quadratic model and *D*_10_ and *D*_50_ values were calculated using the following formulas:

(2) $$D_{10}=\frac{-\alpha +\sqrt{\alpha^{2-2ß\ln\left(0.1\right)}}}{2ß}$$

(3) $$D_{50}=\frac{-\alpha +\sqrt{\alpha^{2-2ß\ln\left(0.5\right)}}}{2ß}$$

### 4.4. AlamarBlue Proliferation Assay

Cell proliferation was measured using the alamarBlue™ Cell Viability Reagent (Thermo Fisher Scientific, Waltham, MA, USA) in a 96-well format. Following lomeguatrib treatment for 24, 48, and 72 h, 10% of the alamarBlue Reagent was added to the cells and incubated for 4 h at 37 °C. Absorbance at 570 nm and 630 nm was determined on a Microplate Reader EL808 (BioTek Instruments Inc., Winooski, VT, USA). Doubling times were calculated using the following formula:

(4) $$Doubling\;Time\;\left(h\right)=\frac{duration\times \log\left(2\right)}{\log\left(final\;absorption\right)-\log\left(inital\;absorption\right)}$$

### 4.5. Western Blot

48 h after lomeguatrib, i.e., 24 h after radiation treatment, cells were lysed in RIPA buffer (150 mM NaCl, 50 mM Tris, 0.1% SDS; 1% Triton X-100, 0.5% sodium deoxycholate, 10× phosphatase inhibitor, 25× protease inhibitor, 1 mM PMSF) on ice for 30 min with vortexing every two minutes. After centrifugation at 12,000 rpm for 10 min at 4 °C protein lysates were collected from the supernatants and stored at −80 °C for further use. Protein concentration was determined using the Pierce™ BCA™ Protein-Assay (Thermo Fisher Scientific, Waltham, MA, USA). Proteins were separated by SDS-PAGE and transferred to nitrocellulose membranes. Incubation with the primary antibodies anti-MGMT (1:200, Santa-Cruz, Dallas, TX, USA) and anti-ß-actin (1:100,000, Sigma-Aldrich, St. Louis, MO, USA) was done overnight at 4 °C. Secondary antibody incubation using the anti-mouse IgG (H+L) AP conjugate (1:10,000, Santa-Cruz, Dallas, TX, USA) was done for 2 h at room temperature. Proteins were detected using the Novex™ AP Chromogenes Substrate (BCIP/NBT) (Thermo Fisher Scientific, Waltham, MA, USA).

### 4.6. Cell Cycle Flow Cytometry

24 h after irradiation, i.e., 48 h after lomeguatrib addition, cells were fixed in −20 °C cold 70% for at least two hours. Propidium iodide (PI) staining solution (0.02 mg mL^−1^ PI (Thermo Fisher, Waltham, MA, USA), 0.15% Triton X-100 and 0.2 mg mL^−1^ DNase-free RNase A) was added to the samples and incubated for 30 min in the dark at room temperature. Acquisition was performed in the BD FACSCalibur™ (Becton, Dickinson and Company, Franklin Lakes, NJ, USA). Cell cycle distribution was analyzed using ModFit LT™ software (Verity Software House Inc., Topsham, ME, USA).

### 4.7. Quantification of Apoptosis

Detection of apoptotic cells was performed using the CellEvent™ Caspase-3/7 Green Flow Cytometry Assay Kit (Thermo Fisher Scientific, Waltham, MA, USA) according to the manufacturer’s instructions. 48 h after radiation treatment, i.e., 72 h after lomeguatrib treatment samples were collected and incubated with the Caspase-3/7 Green Detection Reagent for 40 min at room temperature. Staining of the dead cells was done using the SYTOX AADvanced dead cell stain solution for 5 min at 37 °C. Immediately, FACS analysis was performed in the BD FACSCalibur™ (Becton, Dickinson and Company, Franklin Lakes, NJ, USA) and quantification of apoptotic cells was analyzed in the BD CellQuest™ software.

### 4.8. Statistical Analysis

Mean values were calculated and are presented as ± standard error of the mean (SEM). Differences in mean values between groups were compared using Student’s *t*-test. Two-Way ANOVA was used to evaluate significant differences between cell lines in the colony-forming assay using GraphPad Prism. Probability values of *p* < 0.05 were regarded as statistically significant. In order to ensure reproducibility of the results, each experiment was repeated at least three times.

## Figures and Tables

**Table 1:** Doubling times of glioblastoma cell lines in combination with 0 µM, 1 µM, and 20 µM lomeguatrib. *p*-values were calculated by applying the Student’s *t*-test.

Cell Line	Lomeguatrib (µM)	Doubling Time (h)	*p*-Value
LN18	0	16.4 ± 5.4	
	1	16.1 ± 4.8	0.9541
	20	22.6 ± 5.3	0.3065
T98G	0	16.1 ± 1.8	
	1	25.2 ± 1.8	0.5238
	20	30.6 ± 4.2	0.5135
U118	0	20.1 ± 4.8	
	1	18.0 ± 7.3	0.7425
	20	21.4 ± 9.7	0.8757

**Table 2:** Radiobiological parameters of the three established human glioblastoma cell lines with the addition of 1 µM and 20 µM lomeguatrib.

Cell Line	Lomeguatrib	D_50_ (Gy) ^a^	SER (50%) ^b^	α (Gy^−1^) ^c^	ß (Gy^−2^) ^c^
LN18	0 µM	2.27 ± 0.24	1	0.2353 ± 0.1358	0.0333 ± 0.0192
	1 µM	1.71 ± 0.16	1.36 ± 0.08	0.3974 ± 0.1408	0.0059 ± 0.0029
	20 µM	3.05 ± 0.04	0.76 ± 0.06	0.1517 ± 0.0876	0.0249 ± 0.0144
T98G	0 µM	3.29 ± 0.12	1	0.1264 ± 0.0730	0.0255 ± 0.0174
	1 µM	2.54 ± 0.18	1.30 ± 0.05	0.2439 ± 0.1408	0.0116 ± 0.0067
	20 µM	4.72 ± 0.08	0.70 ± 0.01	0.0456 ± 0.0263	0.0214 ± 0.0123
U118	0 µM	1.67 ± 0.11	1	0.3789 ± 0.1694	0.0246 ± 0.0110
	1 µM	1.36 ± 0.09	1.32 ± 0.12	0.4360 ± 0.2517	0.0581 ± 0.0366
	20 µM	2.51 ± 0.20	0.66 ± 0.08	0.1637 ± 0.0819	0.0474 ± 0.0237

a: D_50_ dose [Gy] required to reduce cell survival to 50%. b: SER (50%) Sensitization enhancement ratio indicates the extent of the sensitizing effect calculated from D_50_(untreated)/D_50_(treated). c: α and ß values calculated from the linear-quadratic equation: ln SF = −α × D − ß × D^2^.

**Table 3:** *p*-values from Cell Cycle analysis (Figure 6) calculated using Student’s *t*-test.

Cell Line	Comparison	*p*-Value		
		G1	G2/M	S
LN18	0 Gy 0 µM–0 Gy 1 µM	0.7216	0.2716	0.5557
	0 Gy 0 µM–8 Gy 0 µM	**<0.0001**	**<0.0001**	0.0957
	0 Gy 1 µM–8 Gy 1 µM	**<0.0001**	**0.0002**	0.3799
	8 Gy 0 µM–8 Gy 1 µM	0.6659	0.9615	0.6854
	0 Gy 0 µM–0 Gy 20 µM	0.8523	0.9020	0.9125
	0 Gy 0 µM–8 Gy 0 µM	**0.0025**	**0.0003**	0.0849
	0 Gy 20 µM–8 Gy 20 µM	**0.0100**	**0.0374**	0.6565
	8 Gy 0 µM–8 Gy 20 µM	**0.0332**	**0.0085**	0.0687
T98G	0 Gy 0 µM–0 Gy 1 µM	0.8923	0.3786	0.8771
	0 Gy 0 µM–8 Gy 0 µM	0.0580	**0.0003**	0.1098
	0 Gy 1 µM–8 Gy 1 µM	**0.0419**	**0.0002**	0.0510
	8 Gy 0 µM–8 Gy 1 µM	0.7003	0.9021	0.8021
	0 Gy 0 µM–0 Gy 20 µM	0.6737	**0.0140**	0.1019
	0 Gy 0 µM–8 Gy 0 µM	**0.0006**	**0.0024**	0.9001
	0 Gy 20 µM–8 Gy 20 µM	**0.0025**	**0.0001**	0.1456
	8 Gy 0 µM–8 Gy 20 µM	**0.0342**	0.0511	0.9310
U118	0 Gy 0 µM–0 Gy 1 µM	0.1068	0.7759	0.8115
	0 Gy 0 µM–8 Gy 0 µM	**<0.0001**	**<0.0001**	**0.0007**
	0 Gy 1 µM–8 Gy 1 µM	**<0.0001**	**<0.0001**	**0.0041**
	8 Gy 0 µM–8 Gy 1 µM	0.6896	0.0941	0.0532
	0 Gy 0 µM–0 Gy 20 µM	0.2950	0.0545	0.6297
	0 Gy 0 µM–8 Gy 0 µM	**0.0047**	**0.0006**	0.2414
	0 Gy 20 µM–8 Gy 20 µM	**0.0124**	**0.0019**	0.1552
	8 Gy 0 µM–8 Gy 20 µM	0.0809	0.2753	0.7805

## Data Availability

Data is contained within the article.

## References

[1] Ostrom Q.T., Gittleman H., Farah P., Ondracek A., Chen Y., Wolinsky Y., Stroup N.E., Kruchko C., Barnholtz-Sloan J.S. (2013). CBTRUS statistical report: Primary brain and central nervous system tumors diagnosed in the United States in 2006–2010. Neuro-Oncology.

[2] Furnari F.B., Fenton T., Bachoo R.M., Mukasa A., Stommel J.M., Stegh A., Hahn W.C., Ligon K.L., Louis D.N., Brennan C. (2007). Malignant astrocytic glioma: Genetics, biology, and paths to treatment. Genes Dev..

[3] Louis D.N., Perry A., Reifenberger G., Von Deimling A., Figarella-Branger D., Cavenee W.K., Ohgaki H., Wiestler O.D., Kleihues P., Ellison D.W. (2016). The 2016 World Health Organization classification of tumors of the central nervous system: A summary. Acta Neuropathol..

[4] Stewart B., Wild C.P. (2019). World cancer report 2014. Public Health.

[5] Fernandes C., Costa A., Osório L., Lago R.C., Linhares P., Carvalho B., Caeiro C. (2017). Current standards of care in glioblastoma therapy. Exon Publ..

[6] Li Y.M., Suki D., Hess K., Sawaya R. (2016). The influence of maximum safe resection of glioblastoma on survival in 1229 patients: Can we do better than gross-total resection?. J. Neurosurg..

[7] Hottinger A.F., Stupp R., Homicsko K. (2014). Standards of care and novel approaches in the management of glioblastoma multiforme. Chin. J. Cancer.

[8] Stupp R., Mason W.P., Van Den Bent M.J., Weller M., Fisher B., Taphoorn M.J., Belanger K., Brandes A.A., Marosi C., Bogdahn U. (2005). Radiotherapy plus concomitant and adjuvant temozolomide for glioblastoma. N. Engl. J. Med..

[9] Roy S., Lahiri D., Maji T., Biswas J. (2015). Recurrent glioblastoma: Where we stand. South Asian J. Cancer.

[10] Newlands E., Stevens M., Wedge S., Wheelhouse R.T., Brock C. (1997). Temozolomide: A review of its discovery, chemical properties, pre-clinical development and clinical trials. Cancer Treat. Rev..

[11] Barciszewska A.-M., Gurda D., Głodowicz P., Nowak S., Naskręt-Barciszewska M.Z. (2015). A new epigenetic mechanism of temozolomide action in glioma cells. PLoS ONE.

[12] Brennand J., Margison G.P. (1986). Reduction of the toxicity and mutagenicity of alkylating agents in mammalian cells harboring the Escherichia coli alkyltransferase gene. Proc. Natl. Acad. Sci. USA.

[13] Mitra G., Pauly G.T., Kumar R., Pei G.K., Hughes S.H., Moschel R.C., Barbacid M. (1989). Molecular analysis of O6-substituted guanine-induced mutagenesis of ras oncogenes. Proc. Natl. Acad. Sci. USA.

[14] Zhang J., Stevens M.F.G., Tracey D.B. (2012). Temozolomide: Mechanisms of action, repair and resistance. Curr. Mol. Pharmacol..

[15] Pegg A., Byers T. (1992). Repair of DNA containing O6-alkylguanine. FASEB J..

[16] Tano K., Shiota S., Collier J., Foote R.S., Mitra S. (1990). Isolation and structural characterization of a cDNA clone encoding the human DNA repair protein for O6-alkylguanine. Proc. Natl. Acad. Sci. USA.

[17] Hegi M.E., Diserens A.-C., Gorlia T., Hamou M.-F., De Tribolet N., Weller M., Kros J.M., Hainfellner J.A., Mason W., Mariani L. (2005). MGMT gene silencing and benefit from temozolomide in glioblastoma. N. Engl. J. Med..

[18] McElhinney R.S., Donnelly D.J., McCormick J.E., Kelly J., Watson A.J., Rafferty J.A., Elder R.H., Middleton M.R., Willington M.A., McMurry T.B.H. (1998). Inactivation of *O*^6^-alkylguanine-DNA alkyltransferase. 1. Novel *O*^6^-(hetarylmethyl) guanines having basic rings in the side chain. J. Med. Chem..

[19] Middleton M.R., Kelly J., Thatcher N., Donnelly D.J., McElhinney R.S., McMurry T.B.H., McCormick J.E., Margison G.P. (2000). *O*^6^-(4-bromothenyl) guanine improves the therapeutic index of temozolomide against A375M melanoma xenografts. Int. J. Cancer.

[20] Pegg A.E., Boosalis M., Samson L., Moschel R.C., Byers T.L., Swenn K., Dolan M.E. (1993). Mechanism of inactivation of human O6-alkylguanine-DNA alkyltransferase by *O*^6^-benzylguanine. Biochemistry.

[21] Quinn J.A., Desjardins A., Weingart J., Brem H., Dolan M.E., Delaney S.M., Vredenburgh J., Rich J., Friedman A.H., Reardon D.A. (2005). Phase I trial of temozolomide plus *O*^6^-benzylguanine for patients with recurrent or progressive malignant glioma. J. Clin. Oncol..

[22] Quinn J.A., Jiang S.X., Reardon D.A., Desjardins A., Vredenburgh J.J., Rich J.N., Gururangan S., Friedman A.H., Bigner D.D., Sampson J.H. (2009). Phase II trial of temozolomide plus *O*^6^-benzylguanine in adults with recurrent, temozolomide-resistant malignant glioma. J. Clin. Oncol..

[23] Dungey F.A., Löser D.A., Chalmers A.J. (2008). Replication-dependent radiosensitization of human glioma cells by inhibition of poly (ADP-Ribose) polymerase: Mechanisms and therapeutic potential. Int. J. Radiat. Oncol. Biol. Phys..

[24] Jue T.R., Nozue K., Lester A.J., Joshi S., Schroder L.B., Whittaker S.P., Nixdorf S., Rapkins R.W., Khasraw M., McDonald K.L. (2017). Veliparib in combination with radiotherapy for the treatment of MGMT unmethylated glioblastoma. J. Transl. Med..

[25] Robins H.I., Zhang P., Gilbert M.R., Chakravarti A., de Groot J.F., Grimm S.A., Wang F., Lieberman F.S., Krauze A., Trotti A.M. (2016). A randomized phase I/II study of ABT-888 in combination with temozolomide in recurrent temozolomide resistant glioblastoma: An NRG oncology RTOG group study. J. Neuro-Oncol..

[26] Bajan S., Hutvagner G. (2020). RNA-Based Therapeutics: From Antisense Oligonucleotides to miRNAs. Cells.

[27] Hong D.S., Kang Y.-K., Borad M., Sachdev J., Ejadi S., Lim H.Y., Brenner A.J., Park K., Lee J.-L., Kim T.-Y. (2020). Phase 1 study of MRX34, a liposomal miR-34a mimic, in patients with advanced solid tumours. Br. J. Cancer.

[28] Kirstein A., Schmid T.E., Combs S.E. (2020). The role of miRNA for the treatment of MGMT unmethylated glioblastoma multiforme. Cancers.

[29] Reinhard J., Eichhorn U., Wiessler M., Kaina B. (2001). Inactivation of *O*^6^-methylguanine-DNA methyltransferase by glucose-conjugated inhibitors. Int. J. Cancer.

[30] Clemons M., Kelly J., Watson A.J., Howell A., McElhinney R., McMurry T., Margison G.P. (2005). *O*^6^-(4-bromothenyl) guanine reverses temozolomide resistance in human breast tumour MCF-7 cells and xenografts. Br. J. Cancer.

[31] St-Coeur P.-D., Poitras J.J., Cuperlovic-Culf M., Touaibia M. (2015). Investigating a signature of temozolomide resistance in GBM cell lines using metabolomics. J. Neuro-Oncol..

[32] Taspinar M., Ilgaz S., Ozdemir M., Ozkan T., Oztuna D., Canpinar H., Rey J.A., Sunguroğlu A., Castresana J.S., Ugur H.C. (2013). Effect of lomeguatrib–temozolomide combination on MGMT promoter methylation and expression in primary glioblastoma tumor cells. Tumor Biol..

[33] Ugur H.C., Taspinar M., Ilgaz S., Sert F., Canpinar H., Rey J.A., Castresana J.S., Sunguroglu A. (2014). Chemotherapeutic resistance in anaplastic astrocytoma cell lines treated with a temozolomide–lomeguatrib combination. Mol. Biol. Rep..

[34] Signorell R.D., Papachristodoulou A., Xiao J., Arpagaus B., Casalini T., Grandjean J., Thamm J., Steiniger F., Luciani P., Brambilla D. (2018). Preparation of PEGylated liposomes incorporating lipophilic lomeguatrib derivatives for the sensitization of chemo-resistant gliomas. Int. J. Pharm..

[35] Pawlik T.M., Keyomarsi K. (2004). Role of cell cycle in mediating sensitivity to radiotherapy. Int. J. Radiat. Oncol. Biol. Phys..

[36] Iliakis G., Wang Y., Guan J., Wang H. (2003). DNA damage checkpoint control in cells exposed to ionizing radiation. Oncogene.

[37] Taylor W.R., Stark G.R. (2001). Regulation of the G2/M transition by p53. Oncogene.

[38] O’Farrell P.H. (2001). Triggering the all-or-nothing switch into mitosis. Trends Cell Biol..

[39] Kastan M.B., Bartek J. (2004). Cell-cycle checkpoints and cancer. Nature.

[40] Giacinti C., Giordano A. (2006). RB and cell cycle progression. Oncogene.

[41] Network C.G.A.R. (2008). Comprehensive genomic characterization defines human glioblastoma genes and core pathways. Nature.

[42] Hall E.J., Giaccia A.J. (2006). Radiobiology for the Radiologist.

[43] Shi Y., Wang Y., Qian J., Yan X., Han Y., Yao N., Ma J. (2020). MGMT expression affects the gemcitabine resistance of pancreatic cancer cells. Life Sci..

